# New Remedies and Results

**Published:** 1906-05-26

**Authors:** 


### RECENT REMEDIES.

### New Local Anæsthetics.

Ever since the introduction of cocaine as a local anæsthetic, certain disadvantages in its employment have been remarked, not the least being its instability and the liability its solutions have to decompose, and the unaccountable idiosyncrasy which prohibits its use in certain individuals. This has led chemists to investigate the properties of other substances closely allied to cocaine chemically, some of which are found to possess the physiological attributes of cocaine without its accompanying drawbacks.

*Methyl-atropine.*—This subject is a synthetic derivative of atropine and is closely allied to cocaine. It differs from atropine in containing pentad instead of triad nitrogen, which in accordance with a general law in this series of bodies mitigates the toxic properties of the drug. Methyl-atropine is easily soluble in water and dilute alcohol. It may be employed (1) as a substitute for atropine to dilate the pupil. In this case a ½ to 1 per cent. solution is instilled, whereby dilatation of the pupil is rapidly obtained, lasting four to eight hours, with hardly any paralysis of accommodation. Its anæsthetic properties are well marked. (2) It may be used also internally for all purposes for which belladonna is usually given, its action is prompt and there are no bad after-effects. (3) It is a valuable substitute for morphia in abdominal cases, parotitis pruritus, tuberculous peritonitis, etc. As an injection the dose is .0001—.0003 gram, or 3—9 minims of a .05-per-cent. solution. Internally it may be used in capsules containing .0015—.0002 gram twice a day, or for a child of seven years .0005 gram may be similarly used. Local injections (subcutaneous) are effective and convenient. The substance is prepared by E. Merk, of Darmstadt, and has been largely used in Germany and Austria.

*Tropacocaine.*—This substance is a derivative of tropeine, the parent substance of atropine, and occurs naturally in the leaves of Java cocoa. For medicinal purposes it is prepared synthetically in a pure state by Merk. It is easily soluble in water. It is as powerful a local anæsthetic when injected subcutaneously as cocaine, but it is only ⅓ as toxic and has no effect upon the pupil. As a local anæsthetic for operations it should be used in .1 or .2 per cent. solutions in sterilised water, with .2 per cent. sodium chloride added. In ophthalmic work, anæsthesia is produced more rapidly than with corresponding strengths of cocaine solutions; it also passes off more rapidly, and a drop or two may be instilled from time to time during the operation. It may be used in inflamed eyes and causes no corneal clouding. A .2 to .5 per cent. solution has also been employed largely in dental surgery; the latter strength is recommended and will produce anæsthesia of the mucous membrane extending downwards to the alveolus and lasting for ten minutes. The solutions have the great advantage of keeping well, and have been known to remain unimpaired for a year and a half.

*Alypin.*—This substance is not a cocaine or atropine derivative, but is a substituted glycerine. Its hydrochloride is a white crystalline powder, easily soluble in water and alcohol, and neutral in reaction. Its solutions may be boiled for five minutes without detriment, and may be combined with adrenalin or antipyrine if desired. Alypin is equal to cocaine as a local anæsthetic, but is much less toxic and produces no effect on the pupil or the calibre of the blood-vessels. It has been largely used in 10-per-cent. solution painted over the mucous membrane of the nose and throat—four or five applications are generally necessary. If it is desired to produce local anæmia also, a single application of cocaine or adrenalin chloride may be added. Solutions of alypin may similarly be used to produce anæsthesia for small operations, and it has many advantages over cocaine for ophthalmic work. It is practically painless where instilled in 1 or 2 per cent. solutions, it produces its effect in a few minutes, and has no effect on the pupil or the ocular tension. Its price is the same as that of cocaine. The manufacturer is Bayer, of Elberfeld.

